# *Anopheles* larval species composition and characterization of breeding habitats in two localities in the Ghibe River Basin, southwestern Ethiopia

**DOI:** 10.1186/s12936-020-3145-8

**Published:** 2020-02-11

**Authors:** Dejene Getachew, Meshesha Balkew, Habte Tekie

**Affiliations:** 1 Department of Biology, Dire Dawa University, P. O. Box 1362 Dire Dawa, Ethiopia; 2 Abt Associates, PMI VectorLink Ethiopia Project, Addis Ababa, Ethiopia; 3 Department of Zoological Sciences, Addis Ababa University, P. O. Box 1176 Addis Ababa, Ethiopia

**Keywords:** *Anopheles* larvae, Darge, Ghibe, Larval habitat, Ghibe river basin, Pools at river edge

## Abstract

**Background:**

Documentation of the species composition of *Anopheles* mosquitoes and characterization of larval breeding sites is of major importance for the implementation of larval control as part of malaria vector control interventions in Ethiopia. The aims of this study were to determine the *Anopheles* larval species composition, larval density, available habitat types and the effects of related environmental and physico-chemical parameters of habitats in the Ghibe River basin of southwestern Ethiopia.

**Methods:**

*Anopheles* larvae were sampled from November 2014 to October 2016 on a monthly basis and 3rd and 4th instars were identified microscopically to species. The larval habitats were characterized based on habitat perimeter, water depth, intensity of light, water current, water temperature, water pH, water turbidity, distance to the nearest house, vegetation coverage, permanence of the habitat, surface debris coverage, emergent plant coverage, habitat type and substrate type.

**Results:**

In total, 9277 larvae of *Anopheles* mosquitoes and 494 pupae were sampled from borrow pits, hoof prints, rain pools, pools at river edges, pools in drying river beds, rock pools, tire tracks and swamps. *Anopheles* larval density was highest in pools in drying river beds (35.2 larvae per dip) and lowest in swamps (2.1 larvae per dip) at Darge, but highest in rain pools (11.9 larvae per dip), borrow pits (11.2 larvae per dip) and pools at river edges (7.9 larvae per dip), and lowest in swamps (0.5 larvae per dip) at Ghibe. A total of 3485 late instar *Anopheles* mosquito larvae were morphologically identified. *Anopheles gambiae* sensu lato was the primary *Anopheles* mosquito found in all larval habitats except in swamps. Temperature at the time of sampling and emergent vegetation, were the most important variables for *Anopheles* mosquito larval density. *Anopheles gambiae* density was significantly associated with habitats that had smaller perimeters, were sunlit, had low vegetation cover, and a lack of emergent plants. Generally, *Anopheles* mosquito larval density was not significantly associated with water pH, water temperature, water turbidity, algal content, and larval habitat depth.

**Conclusion:**

Different species of *Anopheles* larvae were identified including *An. gambiae s.l*., the main malaria vector in Ethiopia. *Anopheles gambiae s.l.* is the most abundant species that bred in most of the larval habitat types identified in the study area. The density of this species was high in sunlit habitat, absence of emergent plants, lack of vegetation near habitat and habitats closer to human habitation. Rainfall plays a great role in determining the availability of breeding habitats. The presence of rain enable to create some of the habitat types, but alter the habitats formed at the edge of the rivers due to over flooding. Controlling the occurrence of mosquito larvae through larval source management during the dry season, targeting the pools in drying river bed and pools formed at the edge of the rivers as the water receded can be very crucial to interrupt the re-emergence of malaria vectors on the onset of rainy season.

## Background

In 2017, malaria affected 219 million globally and killed 435,000 people. Most cases and deaths occurred in the WHO African Region in children under 5 years of age [1]. In Ethiopia it is estimated that about 75% of the total area of the country is malarious [2]. About 58.3 million people lived in areas at risk of malaria in 2013 [3] and in 2015 it was estimated that malaria infected 2.8 million and resulted in 4900 deaths in Ethiopia [4]. Malaria transmission is seasonal and mostly occurs at the end of the main rainy season from June to August and during the small rains from March to April [2, 5].

As in most malaria endemic countries, the most commonly used malaria vector control interventions in Ethiopia are the application of indoor residual insecticide spraying (IRS) and utilization of long-lasting insecticide-treated nets (LLINs), which target the adult stages of malaria vectors [4]. Due to the development of insecticide resistance by the major malaria vector, *Anopheles arabiensis* [5, 6] and drug resistance by the *Plasmodium* species causing malaria [7, 8], implementation of integrated control interventions that target the larval stage could become very important. In addition to adult vector control, malaria transmission can be reduced by suppressing larval densities using appropriate methods based on the type of their breeding habitat [9, 10].

*Anopheles gambiae* sensu lato larvae are more abundant in sunlit, small and temporary habitats with low emergent plants and canopy cover [11]. These habitats may not favour the development of predators and competitors which may feed upon the mosquito larvae [12, 13]. Such habitats may also dry out due to evaporation before the larvae complete their development [14].

For the implementation of malaria vector control interventions, understanding the distribution patterns of *Anopheles* species in specified area is very important [9, 10]. This can be done through identification of larval habitat ecology and larval population dynamics particularly for the control of immature stages [15]. Control of immature stages of malaria vectors can be advantageous because the larvae are concentrated in specific habitats, relatively immobile, and occupy minimal habitat areas compared with adults that can rapidly disperse over large areas [16]. Hence, knowledge of vector distribution and species composition is very important to design effective malaria vector control programmes [17–19]. In addition, understanding the characteristics of larval habitats is also helpful [17, 20]. Thus, the main objective of this study was to determine the species composition and abundance of *Anopheles* larvae and to describe mosquito larval habitats at Ghibe and Darge study sites in Ghibe River basin, southwestern Ethiopia.

## Methods

### Study area

**Fig. 1:**
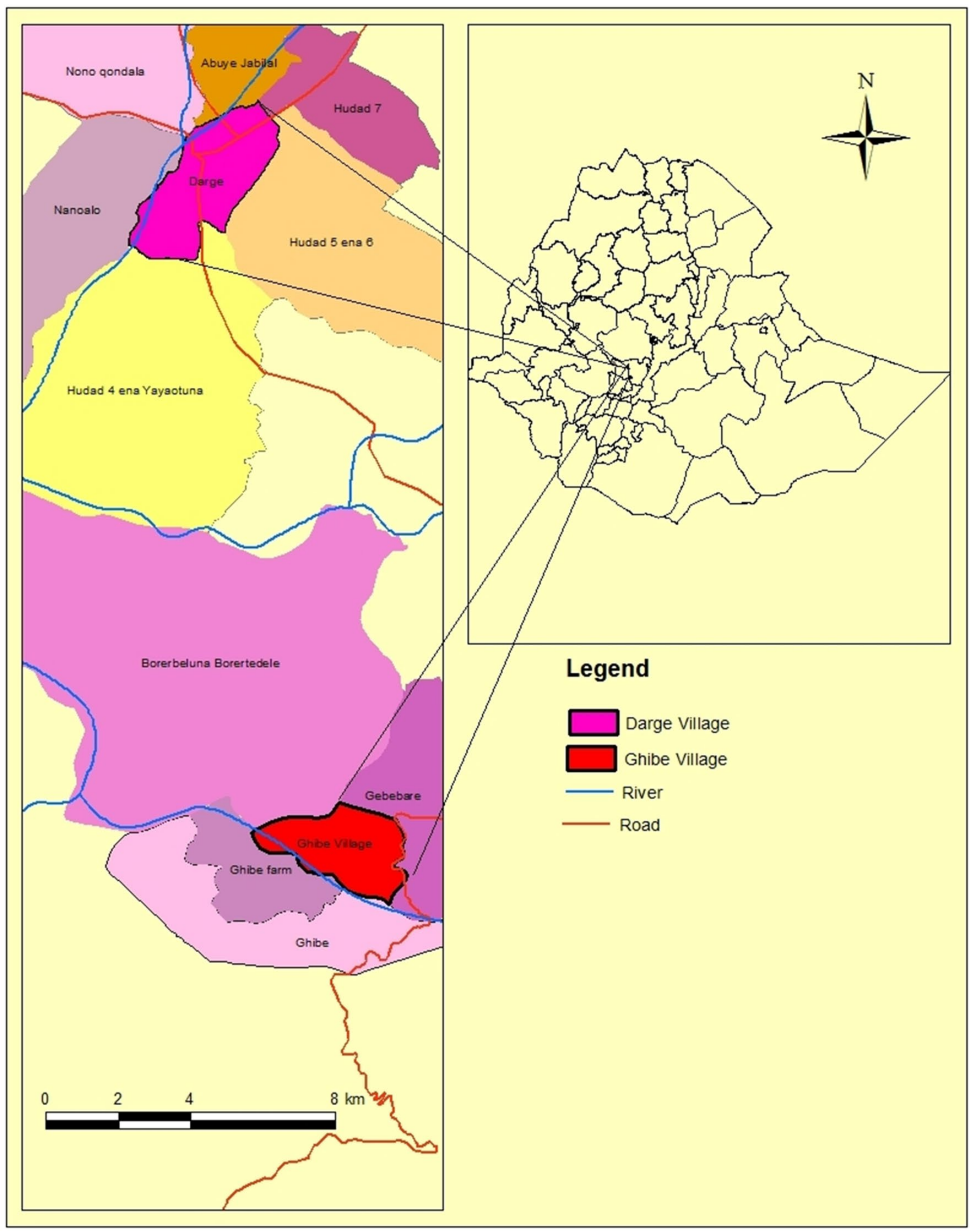
Map of the study area

The study was conducted at Ghibe and Darge study sites located along the Ghibe River basin in southwestern Ethiopia in Abeshge district, Guraghe Zone, Southern Nations Nationalities and Peoples Regional State (Fig. 1). The zonal and Abeshge district town (Wolkite) is located 158 km southwest of Addis Ababa. Ghibe study site [8°14′ N, 37°33′ E, altitude 1080–1134 m above sea level (masl)] is located 30 km south of Wolkite near the Ghibe River. The area has an annual average rainfall of 625 mm (National Meteorological Agency, unpublished report). Acacia trees and savannah grassland dominate the vegetation of the area. In 2016, the study site had 420 households with 2167 total inhabitants of whom 1105 were male and 1062 were female (Abeshge district health office, unpublished report). There is a government-owned health post and one clinic owned by a local Ethiopian seed enterprise.

Darge study site (8°24′ N, 37°31′ E, altitude 1500–1800 masl) is located 42 km west of Wolkite and 52 km from Ghibe study site at the outskirts of Darge town. The Darge River crosses Darge town and serves as one of the tributaries of the Ghibe River. There is a health post and a health center in the study site. In 2016, Darge study site had 731 households with 3518 inhabitants, of whom 1724 were male and 1794 were female (Abeshge district health office, unpublished report). The study site has an annual average rainfall of 1022 mm (National Meteorological Agency, unpublished report). In both study sites, short (March and April) and long (June to August) rainy seasons are important for agricultural activities. The study area was selected due to its malaria endemicity and presence of perennial rivers near the study sites.

### Mosquito larval sampling

Longitudinal larval collections were carried out in each of the study sites every month over a 24-month period (November 2014–October 2016). In each month, mosquito larvae were surveyed in each natural habitat that contained water. During sampling, 3–15 dips were taken using a standard dipper (350 ml capacity, BioQuip Products, Inc. California, USA) depending on the size of each larval habitat at intervals along the edge, with a greater sampling effort in areas of low mosquito density. For small habitats like hoof prints, several sites were pooled to get the required sample volume [13]. The water was collected in a white plastic tray and carefully observed for the presence of *Anopheles* larvae. Sampling was always done in the morning (09:00–12:00) or in the afternoon (14:00–17:00) for about 30 min by the same individual (DG) at each larval habitat.

All *Anopheles* larvae were sorted from culicine larvae and counted. Larval density was determined by taking the average number of mosquito larvae from the total dips taken at specific habitat. *Anopheles* larvae were then sorted into early stages (1st and 2nd instars) and late stages (3rd and 4th instars) and counted and recorded. Early stages were discarded but late stage larvae were killed in hot water (48 to 50 °C for 2 min) and immediately preserved in vials containing 70% ethanol and transported to laboratory for species identification.

### Identification of *Anopheles* mosquito larvae

Third and fourth instar larvae which were collected from different habitats were transported to the Insect Vectors and Entomopathogen Research Laboratory, Department of Zoological Sciences, Addis Ababa University. A drop of Hoyer’s mounting medium was placed on a clean microscopic glass slide. Each larval specimen was mounted on a slide, covered with cover slip and allowed to dry and identified morphologically using the identification key of Gillies and Coetzee [21] under a compound microscope.

### Characterization of larval habitats

Habitats containing *Anopheles* larvae were identified. Environmental variables including habitat perimeter, water depth, direct sunlight, presence of water flow, water temperature at the time of sampling, water pH, water turbidity, distance to the nearest house, vegetation coverage, permanence of the habitat, presence of algae, surface debris coverage, emergent plant coverage, habitat type and substrate type were recorded for each habitat containing *Anopheles* larvae with excluding habitats without larvae.

The depth of water of a habitat was measured from different places depending on size of the habitat using a meter stick and the average depth was taken. The distance to the nearest homestead was measured using a tape measure for less than 100 m and estimated if more than 100 m. Distance was then categorized into four classes: (1) ≤ 100 m, (2) 101 to 200 m, (3) 201 to 300 m, (4) 301 to 400 m. Surface debris, presence of algae and emergent plant coverage were determined based on visual observation. Vegetation cover was visually observed and expressed as open (no vegetation), tree (for the presence of large tree within a range of 10–15 m where shade and foliages could reach), and shrub (woody plants smaller than a tree within 10–15 meters). Habitat perimeter was measured using a tape measure and classified as < 10 m, 10–100 m and > 100 m. Habitat stability was expressed in terms of the length of time the habitat contained water after the rain. A habitat was considered temporary if it held water for 2 weeks or less and permanent if it held water for more than 2 weeks after rain [22]. Though larval sampling was taken on monthly bases, the area was inspected for the presence or absence of rain continuously. Turbidity was measured by placing water samples in glass test tubes and holding them against a white background, and categorized into three levels: low, medium, and highly turbid [23]. Light intensity was visually categorized as sunlit if the habitat received full sunlight that could occur throughout the day, otherwise the site was described as shaded. The substrate type was categorized as mud, stone if the pool was lined with stones that were large in size (rocks generally larger than 10 cm in diameter) and gravel when the stones were small in size but larger than sand. Water temperature was recorded using water thermometer at the time of collection and pH was measured using pH indicator paper [24]. Rainfall of the study area during the study period was obtained from National Meteorological Agency (unpublished report).

### Data analysis

Larval breeding habitats and number of immature *Anopheles* mosquitoes sampled were described using tables. Correlation analysis was used to investigate the relationship between pH, temperature and water depth to the *Anopheles* larval density. *Anopheles* larval density was determined as the number of *Anopheles* larvae (early or late) divided by the number of dips taken from each larval habitat. Larval density was log transformed log_10_ (x + 1) to improve the normality of distribution. Multiple regression analysis was used to identify the environmental variables associated with the occurrence of *Anopheles* larvae. Mann–Whitney *U* test was used to compare samples with two variables; presence of algae (presence or absence), habitat permanency (temporary or permanent), surface debris (present or absent), intensity of light (sunlit or shaded) and water movement (still or flowing). Kruskal–Wallis H test was used to compare samples with more than two groups: water turbidity, water perimeter, distance to the nearest house, canopy cover, emergent plant coverage, habitat type and substrate type. These non-parametric tests were used to compare larval densities from sites with different habitat characteristics.

Data were analysed using IBM SPSS statistical for Windows (IBM corp., Armonk, NY), version 20.0. Values were considered significantly different if p < 0.05 for all the tests.

## Results

### *Anopheles* larvae species composition

*Anopheles* mosquitoes identified from each study site is shown in Table 1. In total, 3485 late instar *Anopheles* mosquito larvae were morphologically identified belonging to 10 species. From the total *Anopheles* larval species, *An. gambiae* sensu lato (*s.l*.), *An. christyi*, *An. pharoensis* and *An*. *pretoriensis* were identified from Darge and Ghibe study sites. *Anopheles gambiae s.l*. constituted 97.8% and *An. pharoensis* 1.3% of all identified larvae in Darge. In Ghibe, 90.6% were *An*. *gambiae s.l*. (henceforth referred to as *An. gambiae*) and 7.9% were *An. christyi*.

**Table 1:** Total number of *Anopheles* larvae identified from Ghibe and Darge study sites (November 2014**–**October 2016)

*Anopheles* larvae identified	Study site	
	Darge *n* (%)	Ghibe *n* (%)
*An. gambiae s.l*.	2317 (97.8)	1012 (90.6)
*An. pharoensis*	30 (1.3)	9 (0.8)
*An. christyi*	13 (0.5)	88 (7.9)
*An. rivulorum*	–	6 (0.5)
*An. demeilloni*	–	1 (0.1)
*An. pretoriensis*	1 (0.0)	1 (0.1)
*An. coustani*	3 (0.1)	–
*An. nili*	1 (0.0)	–
*An. concolor*	2 (0.1)	–
*An. ardensis*	1 (0.0)	–
Total	2368 (100)	1117 (100)

### *Anopheles* larval productivity in different habitat types

The results of larval sampling and the types of larval habitats that were productive in the study area are presented in Table 2. Eight habitat types were identified in Darge, including borrow pits, hoof prints, rain pools, pools at river edges, pools in the bed of drying river, rock pools, tire tracks and swamps. All these types of habitats were also identified in Ghibe except pools in the bed of drying river. In both study sites, the most frequently encountered larval habitats were pools at river edges (Darge, n = 38 and Ghibe, n = 24), rain pools (Darge, n = 24), and tire tracks (Ghibe, n = 17).

**Table 2:** Density of *Anopheles* larvae in different habitat types in Darge and Ghibe study sites (November 2014 to October 2016)

Study site	Habitat type (n)	Total no. of dips	Total larval count	No. of larvae/dip (Mean ± se)	Total pupal count	No. of pupae/dip (Mean ± se)
Darge	Borrow pit (3)	16	228	14.3 ± 8.6	8	0.5 ± 0.1
	Hoof print (8)	52	286	5.5 ± 1.2	8	0.2 ± 0.1
	Rain pool (24)	146	806	5.5 ± 1.5	84	0.6 ± 0.2
	Pools at river edge (38)	231	3063	13.0 ± 2.1	148	0.7 ± 0.2
	Pools in drying	20	704	35.2 ± 7.9	70	3.5 ± 0.8
	river beds (4)					
	Rock pool (7)	42	271	6.5 ± 3.4	27	0.6 ± 0.3
	Tire track (7)	47	300	6.4 ± 3.2	22	0.5 ± 0.2
	Swamp (5)	43	92	2.1 ± 0.2	13	0.3 ± 0.1
	*Total*	*597*	*5750*	*9.6*	*380*	*0.6*
Ghibe	Borrow pit (4)	42	471	11.2 ± 6.3	23	0.5 ± 0.4
	Hoof print (9)	83	328	3.9 ± 0.6	9	0.1 ± 0
	Rain pool (12)	67	800	11.9 ± 5.1	6	0.1 ± 0
	Pools at river edge (24)	145	1146	7.9 ± 1.9	48	0.3 ± 0.1
	Rock pool (4)	20	38	1.9 ± 0.5	0	0 ± 0
	Tire track (17)	138	737	5.3 ± 2.5	28	0.2 ± 0.1
	Swamp (1)	15	7	0.5 ± 0.0	0	0 ± 0
	*Total*	*510*	*3527*	*6.9*	*114*	*0.2*

Values in italics for mean larval and pupal density indicate mean larval or pupal density of each study site

*n* number of larval habitat sampled, *se* standard error of the mean

In total, 9277 larvae and 494 pupae of *Anopheles* mosquitoes were sampled and the result was shown in Table 2. At Darge, large densities of immature *Anopheles* mosquitoes were collected from pools in the drying river beds (mean density of 35.2 larvae per dip and 3.5 pupae per dip). At Ghibe, larvae were densely populated in rain pools, borrow pits and pools at river edges with 11.9, 11.2 and 7.9 larvae per dip and pupae were from borrow pits (0.5 pupae per dip).

### Abundance of *Anopheles* species larvae in breeding sites

*Anopheles* species composition and their preferred habitats in each study site are depicted in Table 3. *Anopheles gambiae* was identified from all types of larval habitats, but *Anopheles christyi* were from pools at river edge, rain pools, rock pools and tire tracks, and *Anopheles pharoensis* were from swamps, borrow pits, pools at river edge and rain pools. In Darge study site, except swamps which had the largest proportion of *An*. *pharoensis* (52.6%), in all other larval habitats almost all the identified larvae were *An. gambiae*. In Ghibe, almost all the identified larvae from hoof prints and tire tracks were *An. gambiae*.

**Table 3:** *Anopheles* mosquito larval species composition in different habitats in the study area (November 2014–October 2016)

Larval species	Habitat type								
	Swamps	Borrow pits	Hoof prints	Pools at river edge	Rain pools	Pools in drying river beds	Rock pools	Tire tracks	Total
Darge									
*An. gambiae* (*s.l*.)	20 (35.1)	151 (100)	67 (100)	1134 (99.3)	362 (99.7)	298 (100)	129 (99.2)	156 (99.4)	2317
*An. christyi*	–	–	–	11 (0.9)	–	–	1 (0.8)	1 (0.6)	13
*An. pharoensis*	30 (52.6)	–	–	–	–	–	–	–	30
*An. coustani*	3 (5.3)	–	–	–	–	–	–	–	3
*An. concolor*	2 (3.5)	–	–	–	–	–	–	–	2
*An. pretoriensis*	–	–	–	–	1 (0.3)	–	–	–	1
*An. nili*	1 (1.8)	–	–	–	–	–	–	–	1
*An. ardensis*	1 (1.8)	–	–	–	–	–	–	–	1
Total	57 (100)	151 (100)	67 (100)	1145 (100)	363 (100)	298 (100)	130 (100)	157 (100)	2368
Ghibe									
*An. gambiae* (*s.l*.)	2 (66.7)	88 (96.7)	179 (100)	381 (80.9)	107 (92.2)	NA	–	255 (99.6)	1012
*An. christyi*	–	–	–	81 (17.2)	5 (4.3)	NA	1 (100)	1 (0.4)	88
*An. pharoensis*	1 (33.3)	3 (3.3)	–	2 (0.4)	3 (2.6)	NA	–	–	9
*An. rivulorum*	–	–	–	6 (1.3)	–	NA	–	–	6
*An. pretoriensis*	–	–	–	–	1 (0.9)	NA	–	–	1
*An. demeilloni*	–	–	–	1 (0.2)	–	NA	–	–	1
Total	3 (100)	91 (100)	179 (100)	471 (100)	116 (100)	NA	1 (100)	256	1117

Values in parenthesis indicate % of total specimens identified in each habitat type

### *Anopheles* species composition and larval habitats

In total, 3485 late instar *Anopheles* mosquito larvae were morphologically identified belonging to 10 species which included *An. gambiae* (95.5%), *An. christyi* (2.9%), *An. pharoensis* (1.1%), *Anopheles rivulorum* (0.2%), *Anopheles coustani* (0.1%), *Anopheles pretoriensis* (0.1%), *Anopheles concolor* (0.1%), *Anopheles demeilloni* (< 0.1%), *Anopheles nili* (< 0.1%) and *Anopheles ardensis* (< 0.1%) (Table 4). In swamps the most abundant species was *An. pharoensis* (51.7%) followed by *An*. *gambiae* (36.7%) and *An. coustani* (5.0%). From borrow pits *An. gambiae* (98.8%) and *An. pharoensis* (1.2%) were identified.

**Table 4:** Late-instar *Anopheles* larvae (and percentage of total for each habitat type) collected in various habitat types in the Ghibe River Basin (November 2014–October 2016)

Larval species				Habitat type					
	Swamps	Borrow pits	Hoof prints	Pools at river edge	Rain pools	Pools in drying river beds	Rock pools	Tire tracks	Total
*An. gambiae* (*s.l*.)	22 (36.7)	239 (98.8)	246 (100)	1515 (93.7)	469 (97.9)	298 (100)	129 (99.2)	411 (99.5)	*3329* (95.5)
*An. christyi*	0	0	0	93 (5.75)	5 (1.04)	0	1 (0.77)	2 (0.48)	*101* (2.90)
*An. pharoensis*	31 (51.66)	3 (1.24)	0	2 (0.12)	3 (0.63)	0	0	0	*39* (1.12)
*An. rivulorum*	0	0	0	6 (0.37)	0	0	0	0	*6* (0.17)
*An. coustani*	3 (5)	0	0	0	0	0	0	0	*3* (0.09)
*An. concolor*	2 (3.33)	0	0	0	0	0	0	0	*2* (0.06)
*An. pretoriensis*	0	0	0	0	2 (0.42)	0	0	0	*2* (0.06)
*An. demeilloni*	0	0	0	1 (0.07)	0	0	0	0	*1* (0.03)
*An. nili*	1 (1.67)	0	0	0	0	0	0	0	*1* (0.03)
*An. ardensis*	1 (1.67)	0	0	0	0	0	0	0	*1* (0.03)
Total	60	242	246	1617	479	298	130	413	3485

Values in parenthesis indicated %

*Anopheles gambiae* was the only species found in hoof prints and pools in drying river beds and the remaining habitats were also identified with large proportions of *An*. *gambiae* larvae. *Anopheles christyi* was identified from pools at river edges, rain pools, rock pools and tire tracks. *Anopheles pharoensis* was collected from swamps, borrow pits, pools at river edges and rain pools (Table 4).

### Association between larval density and habitat variables

The multiple regression model showed that temperature at the time of collection (*p* = 0.03), and emergent vegetation (p = 0.003) were the best predictors of *Anopheles* larval density in the habitats (Table 5). The *F*-ratio in the ANOVA table showed that, the larval densities were statistically significantly associated with the physico-chemical parameters recorded during the larval sampling, *F*_15, 151_ = 3.7, *p* < 0.001, *R*^2^ = 0.266.

**Table 5:** Multiple regression analysis showing the key predicting factors for *Anopheles* larvae density

Variable	B	95% C.I. for B		*t*	Sig.
		Lower	Upper		
Depth	0.006	− 0.002	0.013	1.549	0.123
Temperature	0.027	0.003	0.052	2.197	0.03
pH	0.176	− 0.015	0.367	1.824	0.07
Perimeter	− 0.046	− 0.237	0.145	− 0.475	0.636
Turbidity	− 0.08	− 0.178	0.018	− 1.606	0.11
Distance to nearest house	− 0.071	− 0.163	0.022	− 1.509	0.134
Canopy cover	− 0.068	− 0.234	0.099	− 0.799	0.426
Surface debris	− 0.133	− 0.318	0.051	− 1.429	0.155
Algae	0.114	− 0.069	0.297	1.232	0.22
Emergent plants	− 0.093	− 0.153	− 0.033	− 3.071	0.003
Habitat type	− 0.026	− 0.066	0.014	− 1.278	0.203
Substrate type	− 0.027	− 0.177	0.124	− 0.353	0.725
Intensity of light	0.185	− 0.251	0.622	0.839	0.403
Water current	− 0.584	− 1.302	0.134	− 1.606	0.11
Habitat permanence	0.103	− 0.104	0.31	0.986	0.326
Constant	− 2.301	− 3.913	− 0.689	− 2.82	0.005

*CI* confidence intervals, *B* the unstandardized coefficient value, *Sig* significant at p < 0.05

**Fig. 2:**
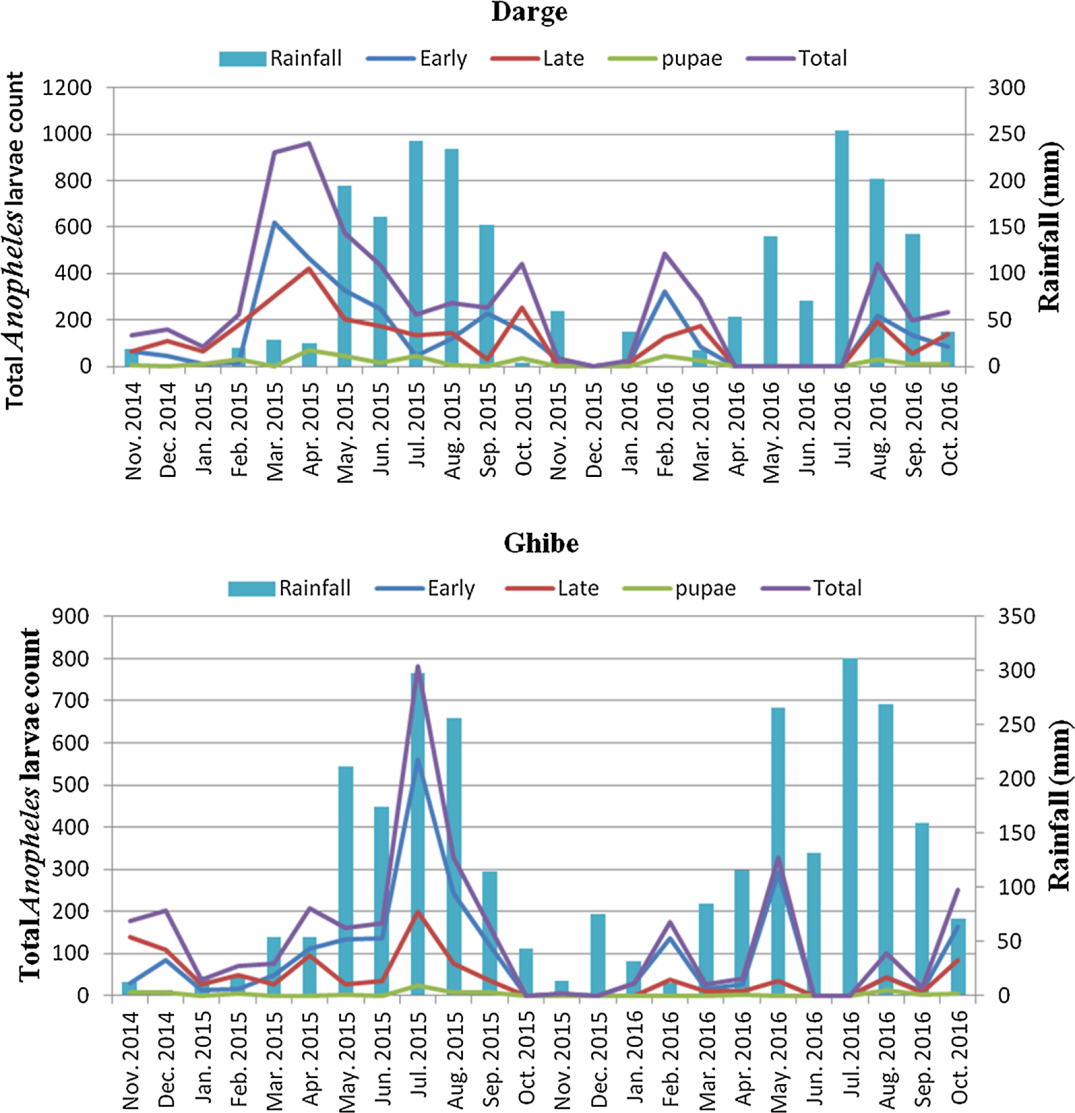
Seasonal distributions of *Anopheles* larvae over the 24-months sampling period

In Ghibe study site, the largest proportion of *Anopheles* larvae were sampled during June–August 2015 and the least were between October 2015–January 2016 and July–August 2016 (Fig. 2). In Darge, during the months of February to May 2015, September to October 2015 and January to March 2016 *Anopheles* larvae were high in number, but they were not collected in December 2015 and between the months of April–July 2016. The study further showed that in Ghibe, early instar *Anopheles* larvae monthly counts were significantly positively correlated with rainfall (r = 0.516, *p* = 0.01) while late instars were not significantly correlated with rainfall (r = 0.114, *p* = 0.595). At Darge, rainfall was not significantly correlated with early (r = − 0.012, *p* = 0.954) or late (r = − 0.104, *p* = 0.629) instar larvae.

There was slight correlation between water pH and that of *Anopheles* larval density (r = 0.187, *p* = 0.016) (Table 6). Water temperature was also positively correlated with the *Anopheles* larval density (r = 0.163, *p* = 0.035). However, there was no association between the depth of the larval habitat and *Anopheles* larval density (r = 0.069, *p* = 0.376).

**Table 6:** Correlation of some larval habitat characteristics with the average *Anopheles* larval density sampled

Physico-chemical parameter	Mean ± SD	Correlation coefficient	*p* value
Water pH	7.19 ± 0.42	0.187*	0.016
Water temperature	28.49 ± 3.24	0.163*	0.035
Water depth	13.49 ± 11.70	0.069	0.376

*SD* standard deviation

*Correlation is significant at the 0.05 level

Adequate numbers of late instar larvae for the analysis of environmental variables were available for *An. gambiae*, *An. christyi* and *An. pharoensis*. Mann–Whitney U tests and Kruskal–Wallis H test showed that (Table 7), *An. gambiae* larval density showed no significant difference between permanent and temporary habitats (*U* = 3137, *p* = 0.455), habitats with or without algae (*p* > 0.05), between habitats of small and large perimeter (p > 0.05), and between clean, moderately turbid, or turbid water (p > 0.05). However, it was significantly higher in habitats without surface debris (*U* = 2117, *p* = 0.009), sunlit habitats (*U* = 228, *p* = 0.028), closer (< 100 m) to human habitations (χ^2^ = 10.6, df = 3, *p* = 0.014), without vegetation (trees or bushes) near larval habitat (χ^2^ = 8.0, df = 2, p = 0.019), and in habitats without emergent plants within the larval habitat (χ^2^ = 8.7, df = 3, *p* = 0.034).

**Table 7:** Environmental variables and distribution of *Anopheles* larvae at Ghibe River basin (November 2014–October 2016)

Environmental factors	*An. gambiae* (*s. l*.)			*An. christyi*			*An. pharoensis*		
	Mean rank	U	*p*	Mean rank	U	*p*	Mean rank	U	*p*
Habitat permanence									
Temporary	81.69	3137	0.455	79.9	2960.5	0.019*	81.46	3115	0.047*
Permanent	87.37			89.96			87.69		
Presence of algae									
Present	90.08	2808	0.235	91.56	2722.5	0.09	84.85	3111.5	0.686
Absent	80.77			79.98			83.55		
Surface debris									
Absent	90.21	2117	0.009*	79.05	2267	0.00*	83.23	2746	0.429
Present	68.6			96.27			85.92		
Intensity of light									
Shade	41.5	228	0.028*	74	423	0.36	93.42	426.5	0.237
Lit	85.58			84.37			83.65		

	*An. gambiae* (*s.l*.)				*An. christyi*				*An. pharoensis*			
	Mean rank	χ^2^	df	*p*	Mean rank	χ^2^	df	*p*	Mean rank	χ^2^	df	*p*
Water perimeter (m)												
< 10 m	85.96	5.17	2	0.075	83.45	2.13	2	0.343	81.3	68.74	2	0.00*
10–100 m	83.3				90.88				83.23			
> 100 m	40.25				74				150			
Turbidity												
Low	87.31	0.58	2	0.748	90.33	5.34	2	0.06	85.71	1.95	2	0.376
Medium	80.54				79.9				85			
High	83.78				80.67				80.69			
Distance to nearest house (m)												
≤ 100	94.62	10.57	3	0.014*	76.04	24.91	3	0.00*	81.14	4.57	3	0.206
101–200	82.36				83.41				88.31			
201–300	63.26				99.83				84.69			
301–400	60.9				116.33				79			
Canopy cover												
Open	89.75	7.955	2	0.019*	84.42	0.454	2	0.797	81.54	9.469	2	0.009*
Tree	64.32				83.36				92.86			
Shrub	83.33				74				79			
Emergent plant												
Absent	93.22	8.65	3	0.034*	82.33	7.54	3	0.057	81.29	11.84	3	0.008*
Grass	71.81				91.57				80.28			
Weeds	89				82.3				86.75			
Grass + weeds	68.9				73.5				95.6			
Substrate type												
Gravel	71.69	2.066	2	0.356	94.44	21.06	2	0.00*	84.03	0.838	2	0.658
Mud	87.37				77.66				84.81			
Stone	78.42				100.24				81.3			

*Statistically significant at *p* < 0.05

*Df* degree of freedom

*Anopheles pharoensis* larval density showed no significant difference in habitats with or without algae (*p* > 0.05), in sunlit and shaded habitats (*p* > 0.05), and between habitats of clean, moderately turbid, or turbid water (p > 0.05). However, larval density was high significantly in permanent than temporary habitats (*U* = 3115, *p* = 0.047), in habitats with perimeter greater than one hundred meter (χ^2^ = 68.7, df = 2, *p* < 0.001), in habitats with the presence of vegetation (χ^2^ = 9.5, df = 2, *p* = 0.009), and in habitats with grass and weeds available together (χ^2^ = 11.8, df = 3, *p* = 0.008).

*Anopheles christyi* larval density showed no significant difference in habitats with or without algae (*p* > 0.05), in sunlit or shaded habitats (*p* > 0.05), between habitats of small and large perimeters (p > 0.05), between habitats of clean, moderately turbid, or turbid water (p > 0.05), and in habitats with or without vegetation (*p* > 0.05). However, larval density was significantly high in permanent habitats than temporary habitat (*U* = 2960.5, *p* = 0.019), with surface debris than without surface debris (*U* = 2267, *p* = 0.00), in habitats that were further (between 301 and 400 m) from houses than closer to houses (χ^2^ = 24.9, df = 3, *p* < 0.001), and in habitats with the substrates that were lined with large stones (χ^2^ = 21.1, df = 2, *p* = 0.00).

## Discussion

This study examined the species composition and identified and characterized the larval habitats of *Anopheles* in two localities in the Ghibe River basin, southwestern Ethiopia. The most commonly encountered larval habitats were pools at river edges, rain pools and tire tracks. During the rainy season water accumulated in sites such as rain pools and tire tracks which served as larval sites. However, these small water bodies do not persist for long if there is no rain [25]. On the other hand, during the rainy season, the rivers increase in size and larval sites may not be formed at the edges of these rivers [26–28]. In line with our study, stream edges served as *Anopheles* breeding habitats during the dry seasons in the central Rift Valley of Ethiopia [24] and in the Butajira area [29].

In Darge, during an extended period without rain, pools of stagnant water formed in the river beds that served as larval breeding habitats. This corroborates with other studies that showed that drying streams [24, 30], drying river beds [25] and habitats at river fringes [27] supported the greatest numbers of mosquito larvae during the dry season. During the dry season, areas which were flooded during the rainy season are very important for the quick re-colonization of the larval habitats shortly after the rainfall [25, 31]. Generally, *Anopheles* larval distribution and abundance are affected by hydrological processes that govern the formation and persistence of different habitat types. Larval productivity depends on rainfall and subsequent changes in water table and river levels [32].

The highest proportions of *Anopheles* larvae were collected from borrow pits, pools in drying river beds and pools at river edges. Larval survival and development depends on biological and physicochemical properties of the habitats [33] including the stability of habitats for longer periods [15], oviposition behavior of gravid females [34], cannibalism and predation by late instars [35, 36], and increased dispersal of early instars by the flow of rivers and streams [37].

Larval breeding habitats such as hoof prints, rain pools, rock pools and tire tracks had lower larval densities than other sites. This corroborates a study conducted in the Butajira area where *Anopheles* larvae were not available in hoof-prints and most temporary rain pools [29]. This could be due to less stable types of habitats (hoof prints, rain pools, and tire tracks) which may dry rapidly after rains [38, 39], and such habitats rarely lasted more than 5 days to enable the larval stages to complete their development to emerge into an adult [15]. It may also have resulted from the infrequent larval sampling which was done on a monthly basis and larvae might not have been missed if it was done on a weekly or fortnightly basis [38]. On the other hand, temporary water bodies such as drainage canals, hoof prints, rain water pools and tire tracks were identified as the most productive habitats in two studies in Kenya [15, 27] but erosion pits and habitats along the river fringe were identified as less important mosquito breeding habitats [27]. This might be related with frequent occurrence of rain in study areas of those reports which support a longer duration of water in these temporary habitats [15].

*Anopheles gambiae* was the primary *Anopheles* mosquito found in all larval habitats except in swamps. In line with this study, *An. gambiae* was found to be the most abundant species in a wide variety of sites in Mbita, western Kenya [40], in stream edges in Eritrea [9] and southern Ethiopia [41] and along river edges during the dry season and short rainy season in the Rift Valley in central Ethiopia [24].

In swamps, the smallest densities of identified *Anopheles* larvae were collected compared to other larval habitats. Sattler et al. [42] stated that *Anopheles* larvae were less likely to be present in swamps and if present they were present in low densities. This type of habitats might support large number of macroinvertebrate predators and competitors which may affect the development of *Anopheles*. The longer development time of *Anopheles* larvae to complete its larval stage in such type of habitats increases their chance to be preyed upon [13, 36]. In swamps, though *An. gambiae* were identified, *An. pharoensis* was the predominant species identified as has been observed elsewhere [24].

Habitats near the edges of rivers were not available when the river level rose as a result of rainfall in upstream areas and, in the case of the Ghibe River, when there was a release of water from Gilgel Gibe I reservoir. When there was intense rainfall the densities of larvae collected was reduced. A study in Eritrea showed that *Anopheles* larval densities were negatively correlated with rainfall [43]. This might happen as the result of the flushing of larvae from habitats and mortality due to heavy rainfall [18, 27, 38].

The study showed that mosquito larvae were abundant after rainy seasons due to the formation of larval habitats, particularly at the edges of rivers which served to sustain *Anopheles* populations during the dry seasons. A study by Gimnig et al. [34] also showed that, the proportion of *Anopheles* mosquito larvae were higher during and immediately after rains. The ability of larval habitats to retain water and the presence of other sources of water during the dry season determines seasonal distribution of *Anopheles* larval development [27].

Temperature at the time of sampling and emergent vegetation, were the most important variables for *Anopheles* larval density. *Anopheles gambiae* density was significantly associated with habitats that had smaller perimeters, were sunlit, had low vegetation cover, and a lack of emergent plants. This is in line with studies conducted elsewhere [24, 34]. Habitats without shade have a higher average daily water temperature than shaded habitats. In habitats with lower water temperature, *Anopheles* larval development period can become elongated and increases the chance of larvae to be predated [44]. In contrast to our study, it was observed that habitats that contained growing grass and other vegetation had more *Anopheles* larvae than habitats without vegetation in study conducted in the Lake Victoria basin, western Kenya [27].

*Anopheles pharoensis* was sampled more from habitats that were permanent with large perimeter, had presence of trees and emergent plants that contain weeds and grasses. A study by Kenea et al. [24] showed that higher densities of *An. pharoensis* larvae were sampled from permanent lakeshore habitats with vegetation and algal mats.

Generally, *Anopheles* larval density was not significantly associated with water pH, water temperature, water turbidity, algal content, and larval habitat depth. The mean water temperature was 28.5 ± 3.2 °C which may have at a range of suitable water temperature for the *Anopheles* larvae to survive and develop into an adult as was stated by Bayoh and Lindsay [45]. Larval habitat depth was measured only from those habitats which contained *Anopheles* larvae so that most of the habitats were not more than 65 cm deep with few exceptions and we did not find that water depth was significantly associated with larval densities. Turbidity was associated with eroded soils that accumulated after rains or when the habitats with muddy substrates became disturbed by animals drinking water from stagnant water particularly at Darge. It has been suggested that turbid habitats attract ovipositing female *Anopheles* [22]. *Anopheles gambiae* larvae were more abundant in habitats closer to human habitation as compared to those habitats far from houses. It was stated that gravid mosquitoes prefer to lay eggs in habitats closer to human habitation to conserve energy lost flying long distances in search of oviposition sites [22].

There were a few limitations to this study. Larval samplings were conducted once each month so the presence of mosquito larvae might be missed during intervening periods which may have affected species abundance and larval habitat productivity. Water temperature of the larval habitat was recorded only during the larval sampling time and was not measured at different times of the day. Characterizations of larval habitats were performed only for those habitats with *Anopheles* larvae. In addition, other environmental variables including a detailed analysis of water chemistry and quantification of mosquito predators and competitors were not done. The important of these factors in mosquito presence and habitat productivity remains unknown.

## Conclusion

*Anopheles gambiae*, which is considered the main malaria vector in Ethiopia was the most abundant species in larval habitats in the study area. Larvae of this species were present during both the dry and rainy seasons. Understanding the breeding habitats of malaria vectors and reducing their availability is important for the control and elimination of malaria [46, 47]. The rivers in the study area served as refugia during the dry seasons, enabling malaria vectors to persist throughout the year. While planning for malaria control programmes that incorporate larval control interventions, both dry and rainy seasons should be considered. As habitats became more limited, application of control interventions during the dry season might be more effective. However, due to the availability of different types of *Anopheles* mosquitoes breeding habitats in the study area, environmental management interventions can be significantly lower mosquito productivity [47].

## Data Availability

The data sets generated and/or analysed during the current study are available from the corresponding author on reasonable request.

## Abbreviations

IRS: Indoor residual insecticide spraying
LLINs: Long-lasting insecticide-treated nets
ITNs: Insecticide-treated mosquito nets
Masl: Metres above sea level
ANOVA: Analysis of variance
